# Oral and Inactivated Polio Vaccine Coverage and Determinants of Coverage Inequality Among the Most At-Risk Populations in Ethiopia

**DOI:** 10.4269/ajtmh.23-0319

**Published:** 2023-09-25

**Authors:** Samson Gebremedhin, Fisseha Shiferie, Dawit A. Tsegaye, Wondwossen A. Alemayehu, Tamiru Wondie, Jen Donofrio, Frank DelPizzo, Kidist Belete, Gashaw Andarge Biks

**Affiliations:** ^1^ School of Public Health, Addis Ababa University, Addis Ababa, Ethiopia;; ^2^ Project HOPE, Ethiopia Country Office, Addis Ababa, Ethiopia;; ^3^ Project HOPE Headquarter, Washington, District of Columbia;; ^4^ Bill & Melinda Gates Foundation, Seattle, Washington;; ^5^ USAID Ethiopia, Addis Ababa, Ethiopia

## Abstract

Combining oral (OPV) and inactivated (IPV) poliovirus vaccines prevents importation of poliovirus and emergence of circulating vaccine-derived poliovirus. We measured the coverage with IPV and third dose of OPV (OPV-3) and identified determinants of coverage inequality in the most at-risk populations in Ethiopia. A national survey representing 10 partly overlapping underserved populations—pastoralists, conflict-affected areas, urban slums, hard-to-reach settings, developing regions, newly formed regions, internally displaced people (IDPs), refugees, and districts neighboring international and interregional boundaries—was conducted among children 12 to 35 months old (*N* = 3,646). Socioeconomic inequality was measured using the concentration index (CIX) and decomposed using a regression-based approach. One-third (95% CI: 31.5–34.0%) of the children received OPV-3 and IPV. The dual coverage was below 50% in developing regions (19.2%), pastoralists (22.0%), IDPs (22.3%), districts neighboring international (24.1%) and interregional (33.3%) boundaries, refugees (27.0%), conflict-affected areas (29.3%), newly formed regions (33.5%), and hard-to-reach areas (38.9%). Conversely, coverage was better in urban slums (78%). Children from poorest households, living in villages that do not have health posts, and having limited health facility access had increased odds of not receiving the vaccines. Low paternal education, dissatisfaction with vaccination service, fear of vaccine side effects, living in female-headed households, having employed and less empowered mothers were also risk factors. IPV–OPV3 coverage favored the rich (CIX = −0.161, *P <* 0.001), and causes of inequality were: inaccessibility of health facilities (13.3%), dissatisfaction with vaccination service (12.8%), and maternal (4.9%) and paternal (4.9%) illiteracy. Polio vaccination coverage in the most at-risk populations in Ethiopia is suboptimal, threatening the polio eradication initiative.

## INTRODUCTION

The Global Polio Eradication Initiative (GPEI) has drastically reduced the incidence of wild poliovirus (WPV), and the disease is on the brink of eradication.^1^ Among the three immunologically distinct WPV strains, type 2 and 3 have been eradicated. Yet WPV type 1 remains endemic in two countries (Afghanistan and Pakistan), and 33 countries including Ethiopia are designated as “outbreak countries,” which means they are at risk for WPV importation or emergence of circulating vaccine-derived poliovirus (cVDPV).^2^ Africa has successfully interrupted the transmission of WPV, but cVDPV remains a major threat due to failure to sustain high vaccine coverage.^3^

Both oral (OPV) and inactivated (IPV) poliovirus vaccines are contributing toward the eradication of poliomyelitis. Oral poliovirus vaccine is highly effective but rarely lead to vaccine-associated paralytic poliomyelitis (VAPP) or cVDPV outbreaks.^4^^,^^5^ Inactivated poliovirus vaccine protects against all the WPV strains, carries no risk of VAPP or cVDPV but has weaker immunogenicity.^4^^,^^5^ In outbreak-prone countries, combining OPV and IPV maximizes the benefits of both and ensures robust protection.^4^^,^^5^ The global Polio Eradication Endgame Plan calls for the introduction of at least one dose of IPV on the preexisting OPV.^6^ Ultimately, OPV will entirely be withdrawn from use and replaced with the IPV.^5^

Ethiopia introduced IPV into the national vaccination schedule in 2015, ahead of the switch from the trivalent (tOPV) to bivalent (bOPV) OPV.^7^ According to the national vaccination schedule, OPV-0 is given at birth, and infants receive OPV-1, -2, and -3 at 6, 10, and 14 weeks of age. Inactivated poliovirus vaccine is also provided at 14 weeks along with OPV-3. Currently, Ethiopia has fully transited to bOPV.

Globally, with the strengthening of the health information system and expansion of large-scale and representative household surveys such as the Demographic and Health Survey (DHS), national- and subnational-level vaccination coverage data are increasingly becoming available for decision-making in low- and middle-income countries (LMICs). However, data remain scarce in at-risk settings such as areas affected by conflict and fragility, remote and inaccessible settings, mobile pastoralist communities, urban slums, internally displaced people (IDP), and refugees. The situation in Ethiopia is also not different from other LMICs.

In line with the goal of the Reach Every District strategy, the Ethiopian government envisions sustaining 90% routine vaccination coverage at the national level and 80% in all districts.^8^ Further, the GPEI requires countries to sustain high coverage (85% or above) within all at-risk settings.^9^ Yet in 2019, only 32% of Ethiopian children received a birth dose of OPV, and 60% and 55% received OPV-3 and IPV, respectively.^10^ The polio vaccination coverage also exposes inequalities or differences in vaccination coverage based on place of residence (urban versus rural), region, household wealth index, and maternal educational status.^10^ In fact, inequality in vaccination coverage is likely the reflection of both supply-side (e.g., inaccessibility to health services) and demand-side (e.g., limited care-seeking due to low socioeconomic status) factors. The recent COVID-19 outbreak and widespread political instability have threatened to roll back the hard-fought gains of the past 2 decades.

Studies from LMICs witnessed pro-rich inequality in childhood vaccine coverage, meaning the service is used disproportionally by better-off households.^11^^–^^15^ Yet few explained the causes of inequality.^13^^–^^15^ Further, most studies measured inequality based on measles or Pentavalent-3 (a combination of vaccines against diphtheria, pertussis, tetanus, hepatitis B, and *Haemophilus influenzae* type B) vaccination coverage, and that of the polio vaccine has not been sufficiently explored. Assessing inequality in IPV–OPV coverage is imperative because high and equitable coverage is required to achieve the GPEI. Inequality studies based on other antigens might not be directly extrapolated to polio because the latter is being delivered through more intense and diverse approaches, including campaigns and other supplementary immunization activities.

A major threat to the GPEI is the inability of endemic and outbreak-prone countries to sustain high vaccination coverage within all at-risk populations.^16^ In countries like Ethiopia, limited information is available on the coverage of dual IPV–OPV. On the basis of data from a nationwide survey that we recently conducted to assess the burden of zero-dose children (children who failed to receive any routine vaccination) in Ethiopia,^17^ we determined the IPV–OPV coverage in disadvantaged settings including pastoralist communities, developing regions, remote districts, conflict-affected areas, urban slums, IDPs, and refugees.

We believe the finding of the study on vaccination coverage and contributors to inequalities in vaccination coverage will help to make polio vaccines accessible to marginalized communities and contribute toward the polio eradication initiative through making data accessible for programmatic decision-making.

## MATERIALS AND METHODS

### Study design and data source.

The data for this study came from a national survey that Project HOPE (Health Opportunities for People Everywhere): The People-to-People Health Foundation, Inc. undertook in mid-2022 with the goal of estimating the burden of zero-dose children in remote settings of Ethiopia. Project HOPE is U.S.-based international NGO working on health development and humanitarian aid. We presented the key findings of the original survey elsewhere.^17^ On the basis of a comprehensive situational analysis that we conducted ahead of this survey, eight partly overlapping domains were considered most at-risk populations for low vaccination coverage in Ethiopia (see Table 1). We also performed subsample analyses for two more domains–districts neighboring international and inter-regional boundaries.

**Table 1:** Most at-risk populations represented in the study, June 2022

No.	Population domains	Description
Primary population domains		
1	Pastoralist population	Populations of the two predominately pastoralist regions (Afar and Somali) of Ethiopia, and other largely pastoralist districts in southern Oromia, SNNP Region, SWPR, and Gambella region
2	Developing regions	The four underdeveloped regions of Ethiopia: Afar, Benishangul Gumuz, Somali, and Gambella
3	Newly-formed regions	Recently established regions of Sidama and SWPR
4	Conflict-affected areas	Districts in Afar, Amhara, Oromia, and Benishangul Gumuz regions that have been affected by armed conflicts in the previous 12 months of the survey
5	Urban slums	Selected slum areas in Addis Ababa and Dire Dawa city administrations, and other four major towns (Adama, Bahir Dar, Harar, Hawassa)
6	Hard-to-reach areas in major regions of Ethiopia	Selected remote districts in Amhara, Oromia and SNNP regions
7	IDPs	IDP centers in Afar, Amhara, Oromia, and Benishangul Gumuz regions
8	Refugees	Somali, Eritrean, and South Sudanese refuges harboring in refugee camps in Somali, Afar, and Gambella regions
Secondary population domains		
9	Districts neighboring international boundaries	Any of the districts included in the first six groups and neighbor international boundaries
10	Districts neighboring inter-regional boundaries	Any of the districts included in the first six groups and neighbor inter-regional boundaries

IDP = internally displaced people; SNNP = Southern Nations, Nationalities, and Peoples; SWPR = South West Peoples’ Region.

### Sampling design.

The sampling design of the study was adopted from 2018 WHO’s immunization coverage cluster survey manual.^18^ The sample size was determined using Cochran’s Single Proportion Sample Size Formula,^19^ assuming 95% confidence level, 5% margin of error, 31% coverage of OPV-3 in developing regions of Ethiopia,^10^ and 10% compensation for possible nonresponse. Consequently, 360 children per population domain were included.

Using secondary analysis of the 2019 Mini DHS survey of Ethiopia,^10^ we assumed that, on average, 12 toddlers aged 12 to 35 months would be available for selection per enumeration area (EA). Thus, for each domain, a minimum of 30 EAs was required. With the intention of having subsample analysis for the four developing regions of Ethiopia, we decided to take 360 samples from each region, maximizing the sample for that domain to 1,440 toddlers. In urban slums, we draw 480, rather than 360, samples due to slightly different statistical assumptions used for sample size computation. Ultimately, we decided to include 4,080 children aged 12 to 35 months from 340 EAs. However, in the actual survey, because of inaccessibility of few EAs secondary to conflict and political instability, we enrolled 3,646 children.

Eligible children were drawn using multistage cluster sampling approach. For the first six domains (excluding refugees and IDPs), based on our preceding situational analysis,^17^ we purposefully selected ∼120 undervaccinated districts. On the basis of the sampling frame developed by the Ethiopian Statistical Service for an upcoming national census, in each district, on average, two EAs were randomly drawn. For urban slums, major shanty villages in Addis Ababa, Adama, Bahir Dar, Hawassa, Harar, and Dire Dawa cities were identified for possible inclusion, and ultimately 40 were randomly selected. In the case of IDPs and refugees, camps were considered as EAs. Ultimately, all eligible children in the EA/village/camp were listed, and 12 selected using smartphone-based random number generator.

### Variables of the study.

The primary outcome of interest for the study was dual IPV–OPV-3 vaccination status dichotomized as “yes” or “no.” Children who had received both OPV-3 and IPV were considered as having dual protection. Key explanatory variables include socioeconomic profile (type of population, household wealth index, maternal and paternal educational status, place of residence), gender-related factors (women domestic decision-making power, maternal employment, sex of the household head, sex of the baby), access to health service (presence of health facility in the kebele, one-way walking distance to the nearest health facility, whether the household had been visited by health extension workers in the past 3 months), and demand-side factors (perceived susceptibility to vaccine-preventable diseases [VPDs], perceived severity of VPDs, knowledge of vaccination, and reported fear of vaccine side effects and vaccine hesitancy).

### Data collection: tools and procedures.

The questionnaire used in the study was developed through expert group discussion and review of relevant literature including the DHS questionnaire. The questionnaire was developed in English and translated into five local languages (Amharic, Afan Oromo, Somali, Afar Af, Sidaamu Afoo), widely spoken in the study localities. The tool was pretested, digitized via the CommCare digital App,^20^ and administered by trained and supervised enumerators. Sociodemographic data were collected using standard questions adapted from the DHS. Household wealth was measured as commonly done in DHS,^21^ based on 41 items related to ownership of valuable household assets and livestock, landholding, materials used for house construction, and access to basic social services.

The vaccination status of the children was determined by triangulating family-held vaccination records, health facility registers, and parent’s recall. Vaccinations provided to the child irrespective of timing and mode of delivery (routine or campaign) were taken into account. In the presence of family-held records, vaccination status was determined based on the same. In case of missing home-based record, the enumerator approached the nearest health post and retrieved the registers of the child from facility-based records. When home- and facility-based records were not available, vaccination status was determined based on caregivers’ reports as is commonly done in DHS. Infants were considered to have received the birth doses of OPV (OPV-0) if they got the vaccine within the first 2 weeks of birth. OPV-1 to OPV-3 status was determined based on the number of oral polio vaccines the child received.

Married women’s decision-making autonomy in household matters was assessed using six-item scale adapted from the DHS questionnaire. The scale measured self-reported autonomy in six spheres of decision-making including making major household purchases, expending own and partner’s income, visiting family or friends, own healthcare, and healthcare for children. Each item was rated as “0” when the decision was solely made by the partner and “1” when the decision was made by the woman or in joint with her partner. Ultimately the scores were summed and classified as low (0–2), medium (3 or 4), or high (5 or 6).

Caregiver’s perceived sense of vulnerability to VPDs and perceived severity of VPD were rated using 4-point Likert scales. The earlier was assessed by asking, “How likely would your child get a VPD if unvaccinated?” and responses were provided as “very likely,” “somewhat likely,” “undecided,” and “not at all.” The latter was assessed by asking, “How serious would it be if your child gets a VPD?” and responses were ordered as “very serious,” “somewhat serious,” “undecided,” and “not at all.”

Caregiver’s knowledge about vaccination was assessed based on three items: awareness about VPDs, where to get vaccination service, and when to initiate vaccination for the child. Participants responded to each item, and those who listed at least three childhood VPDs, knew one or more places to get vaccination service, and reported that vaccination has to be commenced immediately at birth were considered to have comprehensive knowledge about vaccination.

### Data management and analysis.

Data analysis was done using Stata SE, version 16 (StataCorp, College Station, TX). Vaccination coverage estimates were provided separately for the eight population domains. We also performed subsample analysis for two more domains–districts neighboring national and international boundaries. Data were weighted to yield representative aggregated figures. To balance weighted and unweighted sample size, linearization of post-stratification weights was applied.

Wealth index was computed as a composite index of socioeconomic status. The original 41 variables were reduced into nine factors using principal component analysis,^21^ summed into a composite score, and divided into five quintiles (poorest, poorer, middle, richer, richest).

Predictors of IPV–OPV-3 status were identified using generalized estimating equation (GEE) model for correlated data.^22^ Initially, univariable weighted GEE models with logit function were fitted, then models were adjusted for theoretical confounders regardless of empirical evidence.^23^ Model outputs are summarized using crude odds ratio and adjusted odds ratio (aOR).

The concentration curve was used to identify wealth-related inequality in dual IPV–OPV-3 coverage.^16^ Erreygers corrected concentration index (CIX) for binary outcome^24^ was used to quantify the degree of inequality. The CIX would be zero in situations where there is no socioeconomic-related inequality, takes a negative value when the outcome is pro-rich, and becomes positive when its pro-poor.^24^^,^^25^ A regression-based method recommended by Wagstaff and colleagues^24^ was used to decompose the CIX and understand the underlying causes of the inequality.

## RESULTS

### Background characteristics.

A total of 3,646 children aged 12 to 35 months of age were enrolled in the survey. In 93.4% of the cases, mothers of the children provided the data. More than four-fifths (81.4%) of the respondents were drawn from rural areas. The mean (± SD) age of the respondents was 28.7 (± 6.7) years, and 54.0% were between 25 and 34 years of age. More than half had no formal education (59.2%) and were unemployed (56.2%) at the time of the survey. The vast majority (90.8%) of the respondents were married, and about half had partners with no formal education. Approximately one in ten of the households were female-headed. In the survey, boys (54.4%) were slightly overrepresented compared with girls (45.6%), and nearly half of the children (50.7%) were between 12 and 23 months of age. Table 2 summarizes the sociodemographic profile of the study participants and number of respondents drawn from different population domains.

**Table 2:** Sociodemographic characteristics of the study participants, most at-risk populations in Ethiopia, June 2022

Sociodemographic characteristics (*N* = 3,646)	Frequency	Percent
Respondent		
Mother	3,407	93.4
Other primary caregivers	239	6.5
Place of residence		
Urban	677	18.6
Rural	2,969	81.4
Age of the respondent (years)		
15–24	875	24.0
25–34	1,969	54.0
35–44	572	15.7
45 or above	105	2.9
Not sure	126	3.5
Educational status of the respondent		
No formal education or preschool education	2,158	59.2
Primary education	788	21.6
Secondary education	616	16.9
Tertiary education	84	2.3
Marital status		
Married/Living together	3,312	90.8
Widowed, separated, or divorced	291	8.0
Not ever married	43	1.2
Educational status of the partner (*N* = 3,312)		
No formal education or preschool education	1,674	50.5
Primary education	716	21.6
Secondary education	697	21.0
Tertiary education	215	6.5
Not sure	11	0.3
Respondent’s occupation		
Unemployed/housewife	2,048	56.2
Agriculture	1,052	28.9
Trade, including petty trade	272	7.5
Manual (unskilled or skilled)	129	3.5
Others	145	4.0
Gender of the household head		
Male	3,318	91.0
Female	328	9.0
Type of population/study setting*		
Developing regions	1,380	37.8
Pastoralist populations	1,344	36.9
Hard-to-reach areas in major regions	786	21.6
Newly-formed regions	418	11.5
Urban slums	443	12.2
Refugees	311	8.5
Conflict-affected areas	276	7.6
Internally displaced people	264	7.2

* Total exceeds the total sample size (3,336) as some populations contributed to more than one domain.

### OPV and IPV coverage.

**Figure 1.**
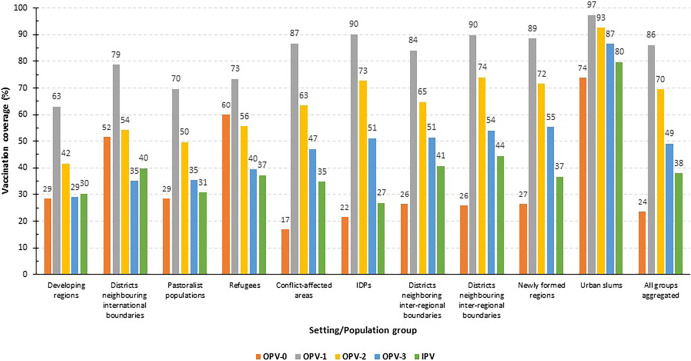
Polio vaccines coverage (%) within most at-risk populations in Ethiopia, June 2022.

Figure 1 presents OPV and IPV coverage within at-risk populations in Ethiopia. Approximately one-quarter (23.7%) received OPV-0, and less than half received OPV-3 (49.1%). OPV-3 coverage was below 50% in developing regions, districts neighboring international boundaries, pastoralist communities, refugees, and conflict-affected areas. Coverages for OPV-1 (86.0%) and OPV-2 (69.5%) were relatively higher. The percentage of children who dropped out of OPV1 to OPV3 vaccination was 17.0%. Approximately one-third of the children (38.0%) received IPV. With the exception of urban slums, in all the domains the coverage was below 50%. Only a quarter of IDPs and about one-third of children from emerging regions, pastoralists, conflict-affected, and newly formed regions received IPV (Figure 1).

### Dual IPV-OPV-3 coverage.

**Figure 2.**
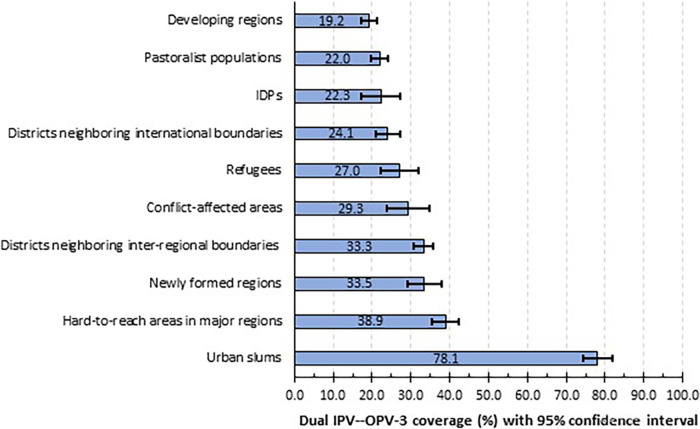
Inactivated poliovirus vaccine and third dose of oral poliovirus vaccine coverage in most at-risk populations in Ethiopia, June 2022.

In aggregate, only 32.5% (95% CI: 31.5–34.0%) of children received both OPV-3 and IPV. Figure 2 shows that dual IPV–OPV-3 coverage was below 25% in developing regions (19.2%), pastoralist populations (22.0%), IDPs (22.3%) and districts bordering international boundaries (24.1%). The IPV-OPV-3 coverage was relatively better in urban slums (78.1%) (Figure 2).

### Socioeconomic differentials of IPV–OPV-3 coverage.

Across the wealth index quintiles, IPV–OPV-3 coverage raised in a dose–response manner from 16% in children from the lowest to 55.4% in the highest quintile. Coverage also increases with improving paternal education but not with maternal education and place of residence (urban/rural). With reference to caregivers who had only one under-5 child in the household, those with three or more children had 1.53 times (95% CI: 1.04–2.27) increased possibility of not getting the vaccines (Table 3).

**Table 3:** Socioeconomic differentials of IPV–OPV-3 coverage in most at-risk populations in Ethiopia, June 2022

Variables	IPV-OPV-3 coverage	COR	aOR*
Household wealth quintile			
Poorest	16.0	2.76 (1.99–3.83)†	2.13 (1.49–3.03)†
Poorer	27.4	1.37 (1.01–1.85)†	1.12 (0.82–1.52)
Middle	32.0	1.26 (0.97–1.64)	1.09 (0.83–1.44)
Richer	38.7	1.02 (0.78–1.32)	0.92 (0.70–1.20)
Richest	55.4	1	1
Maternal education status			
No formal education	25.2	2.84 (1.90–4.25)†	1.38 (0.88–2.17)
Primary education	38.0	2.21 (1.47–3.33)†	1.25 (0.78–2.01)
Secondary education	45.2	1.66 (1.13–2.43)†	1.17 (0.76–1.81)
Tertiary education	39.1	1	1
Partner’s educational status			
No formal education	26.4	2.97 (2.13–4.14)†	2.04 (1.48–2.81)†
Primary education	40.3	2.27 (1.59–3.25)†	1.87 (1.31–2.69)†
Secondary education	41.7	1.97 (1.40–2.77)†	1.81 (1.27–2.57)†
Tertiary education	52.3	1	1
Place of residence			
Rural	28.3	1	1
Urban	49.0	0.41 (0.30–0.57)†	0.83 (0.63–2.00)
Number of under-5 children			
1	35.9	1	1
2	30.7	1.24 (1.01–1.53)†	1.12 (0.90–1.40)
3 or more	22.3	1.84 (1.25–2.71)†	1.53 (1.04–2.27)†

aOR = adjusted odds ratio; COR = crude odds ratio; IPV–OPV-3 = inactivated poliovirus vaccine and third dose of oral poliovirus vaccine.

* Adjusted for all variables presented in the table.

† Significant association at 5% level of significance.

### Access to health service as a determinant of IPV–OPV-3 coverage.

IPV-OPV-3 coverage was lower among children from kebeles (the smallest local administrative unit) not having their own health facilities (aOR: 2.68, 95% CI: 1.59–4.53) and household located more than 1 hour walking distance from the nearby health facility (aOR: 1.42, 95% CI: 1.03–1.97). Caregivers who reported that they were not satisfied with vaccination service (aOR: 2.15, 95% CI: 1.53–3.01) or had not been visited by frontline health workers in the past 3 months (aOR: 1.63, 95% CI: 1.26–2.12) had lower uptake (Table 4).

**Table 4:** Access to health services and IPV–OPV-3 coverage in most at-risk populations in Ethiopia, June 2022

Access to health service	IPV–OPV-3 coverage	COR	aOR*
Walking distance from the nearest health facility			
≤ 30 minutes	36.8	1	1
30–60 minutes	31.9	1.43 (1.16–1.77)†	0.89 (0.67–1.20)
> 1 hour	24.1	1.80 (1.40–2.32)†	1.42 (1.03–1.97)†
Presence of health facility in the kebele/village			
Yes	34.4	1	1
No	22.7	1.81 (2.47–5.90)†	2.68 (1.59–4.53)†
Perceived satisfaction with vaccination service			
Yes	39.7	1	1
No	16.3	3.10 (2.21–4.34)†	2.15 (1.53–3.01)†
Home visit by HEWs in the past 3 months			
Yes	38.7	1	1
No	26.4	1.90 (1.46–2.47)†	1.63 (1.26–2.12)†

aOR = adjusted odds ratio; COR = crude odds ratio; HEW = health extension worker; IPV–OPV-3 = inactivated poliovirus vaccine and third dose of oral poliovirus vaccine.

* Adjusted for all variables in the table, household wealth index, maternal education, and paternal education.

† Significant association at 5% level of significance.

### Demand-side barriers as determinants of dual IPV-OPV-3 coverage.

The vast majority (86.5%) of the caregivers perceived that the child would be “very” or “somewhat” susceptible to VPDs if not vaccinated. Similarly, 81.2% supposed VPDs, including polio, could be severe if the child were infected. Pertaining to knowledge, nearly all (99.4%) know where to get their child vaccinated. However, only 45.1% managed to mention at least three VPD disease, and 19.1% were aware that infants have to be vaccinated starting from birth. Smaller proportions reported fear of vaccine side effects (2.1%) and refused vaccination offered by a health worker (0.4%). Caregivers who did report fear of vaccine side effects had significantly reduced odds of receiving the vaccines (Table 5).

**Table 5:** Demand-side factors and IPV–OPV-3 coverage in most at-risk populations in Ethiopia, June 2022

Demand side factors	IPV-OPV-3 coverage	COR	aOR*
Perceived susceptibility to VPDs if the child was not vaccinated			
Likely or somehow likely	35.1	1	1
Unlikely or unsure	15.7	1.80 (1.09–2.99)†	1.35 (0.74–2.46)
Perceived severity of VPD if the child got ill			
Severe or somehow severe	35.7	1	1
Not severe or unsure	18.5	1.67 (1.16–2.49)†	1.46 (0.94–2.28)
Fear of vaccine side effects			
Yes	18.3	1.96 (1.05–3.57)†	1.96 (1.04–3.07)†
No	35.1	1	1
Reported vaccine rejection			
Yes	21.4	1.81 (0.43–7.57)	1.47 (0.24–8.99)
No	34.8	1	1
Comprehensive knowledge on vaccination			
Yes	35.8	1.06 (0.86–1.31)†	1.11 (0.87–1.41)
No	31.7	1	1

aOR = adjusted odds ratio; COR = crude odds ratio; IPV–OPV-3 = inactivated poliovirus vaccine and third dose of oral poliovirus vaccine; VPD = vaccine-preventable disease.

* Adjusted for all variables in the table, plus household wealth index, maternal and paternal education, place of residence, availability of health facility in the kebele, and walking distance from nearest health facility.

† Significant association at 5% level of significance.

### Gender as a determinant of IPV–OPV-3 coverage.

According to the composite index for measuring married women’s household decision-making power, 72.7% had high and 13.9% medium household decision-making power. Infants born to women with low decision-making power had 3 times increased odds (aOR = 2.93, 95% CI: 2.16–4.00) of not receiving IPV–OPV-3 vaccines than those born to women with high decision-making power. Infants from female-headed households (aOR = 1.72; 95% CI: 1.10–2.79) and having employed mothers (aOR = 1.27; 95% CI: 1.02–1.59) had higher odds of not receiving the vaccines (Table 6).

**Table 6:** Gender-related factors and IPV–OPV-3 coverage in in most at-risk populations in Ethiopia, June 2022

Demand side factors	IPV-OPV-3 coverage	COR	aOR*
Sex of the child			
Boy	33.3	1.06 (0.93–1.21)	1.13 (0.98–1.30)
Girl	34.6	1	1
Women’s household decision-making power			
Low (0–2)	22.9	3.23 (2.39–4.36)†	2.93 (2.16–4.00)†
Medium (3–4)	33.7	1.17 (0.92–1.47)	1.21 (0.95–1.54)
High (5–6)	36.5	1	1
Household head			
Male	33.5	1	1
Female	22.5	1.72 (1.32–2.27)†	1.72 (1.10–2.79)†
Mother of the infant working			
Yes	28.7	1.35 (1.08–1.69)†	1.27 (1.02–1.59)†
No	35.3	1	1

aOR = adjusted odds ratio; COR = crude odds ratio; IPV–OPV-3 = inactivated poliovirus vaccine and third dose of oral poliovirus vaccine.

* Adjusted for all variables in the table, plus household wealth index, maternal education and paternal education, place of residence, availability of health facility in the kebele, and walking distance from nearest health facility.

† Significant association at 5% level of significance.

### Measuring and decomposing socioeconomic inequality in IPV-bOPV-3 coverage.

**Figure 3.**
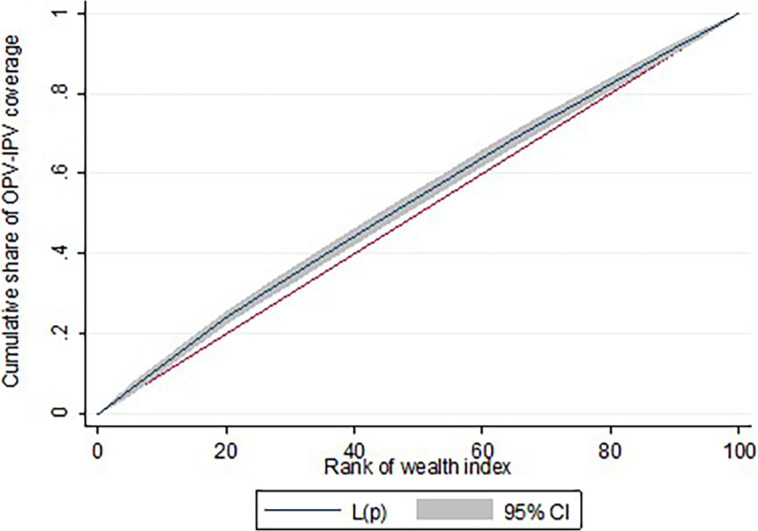
The concentration curve, L(p), in inactivated and third dose of oral poliovirus vaccination coverage in most at-risk populations in Ethiopia, June 2022.

As described earlier, socioeconomic inequality with IPV–OPV-3 vaccination coverage was measured using Erreygers corrected CIX. The CIX was negative (–0.161, *P* < 0.001), implying a pro-rich distribution of service. The concentration curve, L(p), lies above the line of equality suggesting the same (Figure 3).

Decomposing the CIX indicated, absence of a health facility in the kebele (13.3%), dissatisfaction with the vaccination service (12.8%), maternal illiteracy (4.9%), and paternal illiteracy (4.9%) had the highest contribution for the observed inequality (Table 7).

**Table 7:** Decomposition of concentration index for IPV–OPV-3 in remote and underserved settings in Ethiopia

Explanatory variables	Elasticity	Concentration index	Contribution (%)
Absence of health facility in the kebele	−0.683	0.031	13.3
Dissatisfaction with vaccination service	−0.628	0.032	12.8
Maternal illiteracy	0.129	−0.062	4.9
Paternal illiteracy	0.117	−0.067	4.9
Two or more under-5 children in the household	0.158	−0.032	3.2
Household not visited by HEW in the past 3 months	−0.086	0.043	2.3
Absence of health facility within 1-hour walking distance	0.070	0.042	1.8
Low household decision-maker by the caregiver	0.112	−0.011	0.8
Access to mass media	0.009	0.058	0.3
Place of residence (urban vs. rural)	0.010	0.028	0.2

HEW = health extension worker; IPV–OPV-3 = inactivated poliovirus vaccine and third dose of oral poliovirus vaccine.

## DISCUSSION

Polio eradication requires all countries to sustain high vaccination coverage within at-risk populations.^9^ However, the current study indicated that the coverage is largely suboptimal in nearly all at-risk populations, threatening polio eradication efforts in the country and beyond. In Ethiopia, the vaccination coverage within at-risk populations has not been comprehensively studied. The recent DHS-2019 reported that only a quarter of children in the pastoralist regions of Afar and Somali received OPV-3, compared with the national average of 60%.^10^ The pattern for IPV was the same.^10^ A study in pastoralist and semi-pastoralist populations of Ethiopia also indicated, vaccination coverage was improving but low compared with national and global targets.^26^ The IPV–OPV-3 coverage was relatively better in urban slums (78%), although still below the 85% target set by the GPEI.^9^ The higher coverage is likely the reflection of better access to health services in urban areas.

According to DHS, between 2011 and 2019 the national OPV-3 coverage improved from 43% to 60%.^10^^,^^27^ In 2019, IPV coverage stood at 55%.^10^ This suggests that in Ethiopia, low polio vaccination coverage is not only the problem of at-risk populations but also the nation at large. Studies with limited geographic scope witnessed the same.^28^^,^^29^ In the current study, the aggregated coverage of IPV (38%) was lower than that of OPV-3 (48%), even though the two vaccines are expected to be administered concurrently, suggesting missed opportunities within the program. Ethiopia first reported IPV coverage in 2018 based on the routine health information system, 3 years after the introduction of the vaccine. At that time, only 66% of infants received IPV, despite 80% coverage for OPV-3.^30^

OPV at birth ensures early protection against polio in the first few months of life.^31^ Yet with different doses of OPV, the coverage of OPV-0 was exceptionally low (24%). The recent DHS also reported coverage of OPV-0 was 32%—much lower than the uptake of other doses (60%–78%).^10^ The low OPV-0 coverage is likely the reflection of low maternity service utilization in Ethiopia. However, missed opportunities may partly explain the discrepancy. In 2019, the use of skilled attendant at birth (50%) was much higher than the uptake of OPV-0 (32%), suggesting poor integration between maternity and vaccination services.^10^

The study indicated fear of vaccine side effects was relatively rare, but caregivers that reported fear of vaccinating their children had 2 times increased odds of opting out polio vaccine. A study in the high-risk areas of Karachi, Pakistan reported, lack of trust in the polio vaccine and fear of adverse effects were the major reasons for not vaccinating persistently missed children.^32^ Another study from outbreak-prone areas of the same country concluded, refusal of polio vaccination was significantly associated with fear of adverse effects and misconceptions.^33^

Partners’ educational status emerged as a predictor of children’s IPV–OPV-3 status, independent of maternal education or household wealth status, and explained a significant proportion of inequality with polio vaccination. A study based on nationally representative surveys of six LMICs including Ethiopia concluded, children born to fathers having a secondary or above level of education are likely to complete vaccination even if the mother is illiterate.^34^ A study based on national surveys from Afghanistan, Pakistan, and Nigeria also concluded that fathers’ formal education reduces the risk of no or incomplete polio vaccination of children.^35^ The finding suggests that paternal education is equally important as maternal education in improving vaccination outcomes in the study settings.

The study indicated that families having three or more under-5 children are at increased odds of not receiving OPV–IPV independent of other socioeconomic factors including maternal and paternal education. Studies from Pakistan^36^ and the Gambia^37^ also came up with parallel conclusions. Having multiple children restricts the time and resources available to care for children and may result in poor health-seeking behavior.

So far studies have not documented meaningful sex disparities in vaccination coverage,^38^ and we also noted the same. However, other gender-related factors appear to have limited uptake of polio vaccine. Our findings indicated that children from female-headed households, and those having employed and less empowered mothers had lower odds of receiving polio vaccine. Possible explanations may include that working mothers usually have little time to care for their children, female-headed households may have lower care-seeking due to increased domestic workload and lower incomes. Further, poor household decision-making power compromises care seeking behavior. A study in Democratic Republic of the Congo reported that infant born to women empowered on domestic matters had nearly 2 times increased odds of completing vaccination.^39^ A systematic review concluded that in LMICs women’s agency, as measured by domestic decision-making, was positively associated with vaccination service utilization.^40^ According to a multicounty vaccine inequality analysis, vaccination coverage was significantly lower in female- than male-headed households in Ethiopia, but not in Uganda, India, and Nigeria.^41^

The study witnessed significant socioeconomic inequality with IPV–OPV-3 coverage to the advantage of the rich. Furthermore, limited access to health services, dissatisfaction with vaccination service, and maternal and paternal illiteracy underline the inequality. The study might even underestimate the extent of the inequality because it was restricted to disadvantaged communities. Previous studies also witnessed pro-rich inequality in routine childhood vaccination coverage in LMICs,^11^^–^^15^ but few explained the causes of the inequality.^14^^,^^15^ Secondary analysis of 46 DHS in LMICs indicated, vaccination coverage was pro-rich in many countries (median CIX = 0.161), excluding three countries (Gambia, Namibia, and Kyrgyz Republic) where a pro-poor distribution was observed.^11^ In Nigeria, maternal illiteracy, region, place of residence and economic status explained the disparities in vaccination coverage.^14^ In India, the largest contributors to socioeconomic inequality of vaccination coverage were household wealth, institutional delivery, and maternal education.^13^

The findings should be interpreted with consideration of the scope, strength, limitations of the study. To the best of our knowledge, this is the first study that documented polio vaccination coverage within multiple at-risk populations. The sample size was also large to allow subsample analysis for all domains. In terms of scope, the study was deliberately limited to needy population groups hence findings should not be extrapolated to the entire nation. Due to active conflict at the time of the survey, Tigray region and specific districts in Benishangul Gumuz and western Oromia have not been included in the study, and this may somehow limit the generalizability of the study. Causal claims should be cautiously taken, given the cross-sectional design. In addition, we estimated IPV–OPV-3 coverage without taking the sequence and timing of vaccination into consideration.

## CONCLUSION

The study concluded that polio vaccination coverage in most at-risk areas in Ethiopia is largely suboptimal threatening the national and global polio eradication initiative. As long at-risk areas have low vaccination coverage, children across the country and beyond remain at risk of polio. Apart from poor access to health service, polio vaccination is constrained by demand-side factors such as low socioeconomic status, dissatisfaction with vaccination service, and fear of side effects. Polio vaccination also exhibited a pro-rich distribution underlined by similar factors.

To keep pace with the GPEI, Ethiopia needs to strengthen the routine vaccination program in remote areas by rolling out periodic intensification of routine immunization. Furthermore, outreach and supplementary immunization activities such as campaigns have to be strengthened so as to bring the vaccine closer to the most at-risk communities. Robust integration of immunization with other services is also indispensable to reduce missed opportunities. Addressing parents’ concern over vaccine side effects through integrating counseling activities into routine immunization programs is needed. The pro-rich distribution of the vaccination program calls for closing subnational vaccine equity gap in the country. The polio vaccination program should also be gender responsive to engage husbands more effectively and become more responsive to female-headed households and used mothers.
